# Is there a U-shaped relationship between physical activity in leisure time and risk of chronic low back pain? A follow-up in the HUNT Study

**DOI:** 10.1186/s12889-016-2970-8

**Published:** 2016-04-11

**Authors:** Ingrid Heuch, Ivar Heuch, Knut Hagen, John-Anker Zwart

**Affiliations:** Department of Neurology and FORMI, Oslo University Hospital, N-0407 Oslo, Norway; Department of Mathematics, University of Bergen, Bergen, Norway; Department of Neuroscience, Norwegian University of Science and Technology, Trondheim, Norway; Department of Neurology, Norwegian National Headache Centre, St. Olavs Hospital, Trondheim, Norway; Faculty of Medicine, University of Oslo, Oslo, Norway

**Keywords:** Low back pain, Physical activity, Leisure time, U-shape, Epidemiology, Public health

## Abstract

**Background:**

Physical activity in leisure time is often considered to have favourable effects on the risk of low back pain (LBP), but demonstrating a definite association in epidemiological studies has proven difficult. The purpose of the present study was to explore associations between physical activity and risk of chronic LBP in an adult population and to investigate whether relationships are limited to certain age groups or to females or males. A particular objective was to determine whether support could be found for a U-shaped relationship, with both low and high activity levels carrying greater risk.

**Methods:**

The relationship between physical activity and risk of chronic LBP was examined in a Norwegian prospective study using data from the community-based HUNT2 and HUNT3 surveys. Participants were 9616 women and 8452 men without LBP at baseline, who reported after 11 years whether they suffered from LBP. Associations between baseline physical activity in leisure time and risk of chronic LBP at end of follow-up were evaluated by generalized linear modelling with adjustment for potential confounders.

**Results:**

Significant associations between leisure time physical activity and risk were observed in both sexes after age adjustment, mainly suggesting inverse relationships. Women participating in hard physical activity 1–2 h per week had a relative risk (RR) of chronic LBP of 0.81 (95 % CI 0.71–0.93) compared to those with only light physical activity less than 1 h per week. The corresponding RR in men was 0.71 (95 % CI 0.60–0.85). After adjustment for education, employment, occupational activity, body mass index (BMI) and smoking, significant relationships could only be demonstrated in those aged 50 years or more at baseline. The associations differed between female educational groups, with more U-shaped relationships being observed among women with basic education only.

**Conclusion:**

No strong support was found overall for U-shaped relationships. However, no further general decrease in risk was seen among those with 3 h or more of hard physical activity per week. The contrasts observed between female educational groups may reflect different preferences regarding specific strenuous activities. Men aged 50 years or more seem in particular to benefit from hard physical activities.

## Background

Physical inactivity is considered an important factor contributing to the overall burden of disease [1]. Low back pain (LBP) represents a considerable health problem, causing more global disability than any other condition [2]. The general relationship between physical activity and LBP is still uncertain, however, despite the large number of studies that have been carried out [3]. Some reports indicate that patients with high levels of pain and disability due to LBP are less likely to be engaged in physical activity than healthy individuals [4], while other studies suggest that the actual activity pattern may differ [5]. Physical activity does not generally seem to predict future disability or pain levels in LBP patients [6], although such activity may improve prognosis in particular patient categories [7].

Various review papers [3, 8–10] have dealt with associations between physical activity in leisure time and future risk of LBP. Some reviews [3, 8] have pointed out that the evidence from different studies is inconsistent and that no firm conclusion about possible associations can be formulated at present. Specific strenuous sports activities may still be associated with an increased risk of LBP [8]. Occupational physical activities have been considered in many studies, and particular types of work-related stress seem to confer a higher risk of LBP [8, 10], although the overall evidence in support of a causal relationship is not very strong [11]. The direction of the potential relationships with risk of LBP may differ between activities at work and in leisure time, with heavy work loads conferring a higher risk, whereas extensive leisure time physical activity may be more favourable [12].

It has been pointed out [13] that simple dichotomization of physical activity according to a specified criterion may conceal important U-shaped relationships with risk of LBP [14]. Such U-shaped associations with the prevalence of chronic LBP were found, in particular for women, in a relatively large cross-sectional population-based study from the Netherlands [13]. A similar U-shaped relationship was also observed in men in a cross-sectional Korean study [15]. Such results need confirmation in longitudinal studies, however, to shed some light on the underlying causes of LBP, and it is important to check whether the relationships are found consistently in categories defined by age and sex [16].

The purpose of the present study was to explore associations between physical activity in leisure time and the risk of chronic LBP in an adult population, using data collected prospectively in a Norwegian county. None of the participants included in the analysis were suffering from chronic LBP at baseline. A particular objective was to determine whether the data provided support for a U-shaped relationship, and if so, to investigate whether the relationship was limited to certain age groups or to females or males. The same data set has previously been used to study associations between risk of LBP and body mass index (BMI) [17], body height [18], blood pressure [19] and lipid levels [20].

## Methods

### Study population

Three consecutive health surveys have been carried out in the county of Nord-Trøndelag in Norway [21], HUNT1 in 1984–1986, HUNT2 in 1995–1997 and HUNT3 in 2006–2008. The present work is based on data from the HUNT2 survey combined with follow-up data from HUNT3.

All residents of the county who were at least 20 years old received an invitation to take part in the HUNT2 survey. Participants were asked to fill in a questionnaire on health status [22], with one question phrased in this way: “During the last year, have you had pain and/or stiffness in your muscles and limbs that has lasted for at least 3 consecutive months?” If a respondent answered in the affirmative, the following question was asked: “Where did you have pain and/or stiffness?” The lower back was one site listed among several possibilities. Respondents checking this alternative were regarded as suffering from chronic LBP [23]. The participants were also invited to a clinical consultation. Of the 28,906 female and 30,022 male residents in the age interval 30–69 years, 23,205 women and 21,442 men indicated whether they suffered from chronic LBP and gave information on physical activity in leisure time. This corresponds to a participation rate of 75.8 %.

Similar information from a questionnaire and a clinical examination was collected in the HUNT3 survey, carried out 11 years later with a corresponding target population. In the current prospective study, baseline data from HUNT2 were considered in combination with information from HUNT3 on LBP status, in addition to information about residence status supplied by national registries. The information was linked using the unique Norwegian personal identification numbers.

The follow-up in this study aimed at the cohort consisting of 31,145 individuals without chronic LBP aged 30–69 years when they participated in the HUNT2 survey, with information available on physical activity in leisure time. Participants outside this age range in HUNT2 were not included because of relatively low participation rates in the subsequent HUNT3 study. During follow-up, from HUNT2 to HUNT3, 1621 individuals in this cohort died, 1158 left Nord-Trøndelag and one person disappeared. Furthermore, 10,297 members of the cohort resident in the county at the time of HUNT3 did not participate or did not supply information about LBP. Thus a total of 18,068 individuals, 9616 women and 8452 men, were available for analysis after follow-up, representing 63.7 % of the remaining individuals resident in the county and 58.0 % of the original cohort.

### Physical activity in leisure time

A section of the questionnaire used in HUNT2 dealt with physical activity in leisure time during the last year, including moving to and from work. One question was restricted to light activity, defined as activity that did not involve sweating or breathlessness. Another question referred to hard physical activity, causing sweating or shortness of breath. In the present study, five categories of physical activity in leisure time were considered. The first category represented those who reported only light physical activity of duration <1 h per week, and the second represented those who reported only light physical activity ≥1 h per week. The remaining categories included participants reporting at least some hard physical activity, regardless of whether light activity was specified or not. Thus the third, fourth and fifth categories represented hard physical activity of duration <1 h per week, 1–2 h per week and ≥3 h per week, respectively.

### Covariates

Four main categories of work status were defined, the first comprising persons being employed or carrying out professional work. This category was further subdivided according to the level of physical activity at work into four subcategories representing substantially sedentary work (e.g., assembly or desk work), work involving extensive walking but no heavy lifting (e.g., light manufacturing, salespeople or teachers), work leading to both walking and lifting (e.g., postmen, nurses or construction workers) and work involving particularly strenuous activities (e.g., people involved in heavy agricultural or forestry work and heavy construction work). The second main category of work status included those temporarily out of work, students and individuals in military service. The third category included pensioners and people receiving social security support, and the fourth category represented women occupied full-time with housework.

Baseline age was categorized into 10-year intervals. Education was grouped according to duration as ≤9, 10–12, or ≥13 years. BMI, defined as weight/height^2^ and computed in kg/m^2^, was subdivided into three groups: <25, 25–29.9, ≥30. Categories of cigarette smoking represented current daily smoking, previous daily smoking and never daily smoking.

### Data analysis

Associations between baseline physical activity in leisure time and risk of chronic LBP at end of follow-up were evaluated by generalized linear modelling for binomially distributed data with a log-link, including adjustment for potential confounders. All analyses were carried out separately for women and men.

The main analyses were performed with a categorical classification of physical activity in leisure time, with the reference category comprising those who reported light physical activity <1 h per week and no hard physical activity. In separate analyses the successive categories of leisure time physical activity were assigned scores 1, 2, 3, 4, 5, with the model incorporating a linear effect of this score. To explore a possible U-shape in the relationship with risk, particular analyses were also carried out including a quadratic effect of the score in addition to the linear effect.

Initial analyses incorporated adjustment for age only. Additional adjustment was then introduced for other potential risk factors for LBP, as education [24], work status and activity at work [11], BMI [17] and smoking [25]. All variables adjusted for were regarded as categorical. Separate tests were performed for categorical interaction between physical activity in leisure time and each adjustment variable. Because of missing information on potential confounders in a minor part of the data set, analyses with complete adjustment were based on a slightly lower number of individuals than the age-adjusted analyses. All statistical analyses were carried out using IBM SPSS version 21 (IBM Corp., Armonk, New York).

## Results

In both sexes the percentage of chronic LBP at end of follow-up diminished with increasing levels of baseline physical activity in leisure time, except in the category with hard physical activity of duration ≥3 h per week (Table 1). Women in this category had almost the same percentage of chronic LBP as those who only engaged in light physical activity ≥1 h per week. Men who reported hard physical activity ≥3 h per week experienced about the same percentage of LBP as those who participated in hard physical activity <1 h per week (Table 1).

**Table 1:** Proportion of individuals with chronic LBP at end of follow-up, by baseline physical activity

Physical activity in leisure time (hours per week)		Women		Men	
Light	Hard	Total	With chronic LBP at end of follow-up (%)	Total	With chronic LBP at end of follow-up (%)
<1	0	1526	331 (21.7)	1367	220 (16.1)
≥1	0	3629	735 (20.3)	1936	296 (15.3)
	<1^a^	2100	419 (20.0)	2075	295 (14.2)
	1–2	1831	322 (17.6)	1924	221 (11.5)
	≥3	530	108 (20.4)	1150	157 (13.7)

*LBP* low back pain

^a^Must have reported some hard activity

Generalized linear modelling with adjustment for age revealed associations with the same kind of shape (Table 2). Both women and men showed statistically significant categorical associations between leisure time physical activity and risk of chronic LBP. Despite a modest increase in estimated risk comparing those participating in hard physical activity ≥3 h per week with those spending 1–2 h per week, the overall risk pattern reflected a significant decreasing relationship with the score for physical activity in both sexes. Additional quadratic terms did not provide significant contributions.

**Table 2:** Associations between baseline physical activity and risk of chronic LBP

Physical activity in leisure time (hours per week)			Women		Men	
			Adjustment for age	Additional adjustment^a^	Adjustment for age	Additional adjustment^a^
		Total	9616	9199	8452	8130
Light	Hard	Score	RR (95 % CI)	RR (95 % CI)	RR (95 % CI)	RR (95 % CI)
<1	0	1	1.00 (reference)	1.00 (reference)	1.00 (reference)	1.00 (reference)
≥1	0	2	0.94 (0.83–1.05)	0.97 (0.86–1.09)	0.96 (0.82–1.13)	1.03 (0.87–1.21)
	<1^b^	3	0.92 (0.81–1.05)	0.99 (0.87–1.13)	0.88 (0.75–1.03)	0.96 (0.82–1.14)
	1–2	4	0.81 (0.71–0.93)	0.89 (0.77–1.03)	0.71 (0.60–0.85)	0.81 (0.68–0.97)
	≥3	5	0.94 (0.77–1.14)	1.05 (0.86–1.27)	0.85 (0.70–1.03)	0.96 (0.79–1.17)
*P,* categorical effect			0.045	0.36	0.001	0.06
*P*, linear effect^c^			0.016	0.46	0.001	0.07
*P*, quadratic effect^d^			0.39	0.56	0.33	0.69

*LBP* chronic low back pain, *RR* relative risk, *CI* confidence interval

^a^Adjustment for age, education, work status, physical activity at work, BMI, smoking

^b^Must have reported some hard activity

^c^For linear effect of score for physical activity

^d^For quadratic effect of score for physical activity, in model also including linear effect

After additional adjustment for education, work status, physical activity at work, BMI and smoking, significant associations with physical activity in leisure time were no longer seen in the total data set, although categorical and linear effects nearly reached significance in men (Table 2). With this adjustment, the estimated relative risk in women who engaged in hard physical activity ≥3 h per week increased and exceeded the value in the reference group representing those participating in light physical activity <1 h per week only. Associations in women and men did not differ significantly (*P* = 0.55).

Significant interactions with physical activity in leisure time were found among women for the two factors age (*P* = 0.03 with subdivision into broad 20 year intervals) and duration of education (*P* = 0.002), with complete adjustment for potential confounders. No significant interactions were observed among men (with, in particular, *P* = 0.41 for age in 20 year intervals and *P* = 0.49 for education). There was no indication that associations with physical activity differed between categories of BMI (*P* = 0.57 for interaction in women and *P* = 0.98 in men) or occupational categories (*P* = 0.44 in women and *P* = 0.97 in men).

Table 3 shows fully adjusted relative risk estimates for combinations of leisure time physical activity and 20 year age intervals in women and men. The reference category included in each case 30–49 year old individuals with only light physical activity <1 h per week. In addition, separate tests for association with physical activity were carried out within each 20 year age interval. Statistically significant associations were seen in the 50–69 year age intervals but not the 30–49 year intervals. A weak decreasing trend in risk estimates was generally observed in both sexes with increasing physical activity, although the estimated risk of LBP for those participating in hard physical activity ≥3 h per week was still greater than the estimate for hard activity 1–2 h per week.

**Table 3:** Risk of chronic LBP by combinations of age (in 20 year groups) and physical activity^a^

Physical activity in leisure time (hours per week)			Women		Men	
			30–49 years	50–69 years	30–49 years	50–69 years
		Total	5723	3476	4759	3371
Light	Hard	Score	RR (95 % CI)	RR (95 % CI)	RR (95 % CI)	RR (95 % CI)
<1	0	1	1.00 (reference)	1.16 (0.95–1.43)	1.00 (reference)	0.79 (0.61–1.03)
≥1	0	2	1.07 (0.91–1.26)	0.99 (0.83–1.18)	0.95 (0.77–1.18)	0.88 (0.71–1.11)
	<1^b^	3	1.05 (0.89–1.25)	1.08 (0.88–1.34)	0.96 (0.78–1.17)	0.77 (0.60–0.99)
	1–2	4	1.03 (0.86–1.23)	0.73 (0.55–0.95)	0.80 (0.64–1.00)	0.65 (0.50–0.85)
	≥3	5	1.18 (0.93–1.50)	0.99 (0.70–1.41)	1.03 (0.81–1.30)	0.66 (0.48–0.90)
*P,* categorical effect ^c^			0.69	0.006	0.22	0.11
*P,* linear effect^d^			0.33	0.007	0.55	0.030
*P,* quadratic effect^e^			0.92	0.69	0.22	0.36

*LBP* low back pain, *RR* relative risk, *CI* confidence interval

^a^Adjustment for education, work status, physical activity at work, BMI, smoking

^b^Must have reported some hard activity

^c^For categorical effect of physical activity, within 20 year age interval

^d^For linear effect of score for physical activity, within 20 year age interval

^e^For quadratic effect of score for physical activity, within 20 year age interval

Risk estimates for combinations of physical activity in leisure time and duration of education in women are displayed in Table 4. Risk estimates tended to be lower with a longer duration of education. Within groups defined by education, a significant categorical association between leisure time physical activity and risk was observed both for education lasting ≤9 years and ≥13 years. The two associations had a different shape, however, with significant contributions from quadratic terms in the score for physical activity. For education lasting ≤9 years, a U-shape was indicated, but for education lasting ≥13 years, the shape was more similar to an inverted U (or rather an inverted J). Thus the relative risk estimate 1.18 for hard activity ≥3 h per week with an education ≤9 years was the largest one in the entire table, although the corresponding estimate 0.51 with an education ≥13 years was the lowest one. The risk pattern for women with 10–12 years of education was intermediate. Further analysis subdividing data according to both age and education was not practicable because of relatively large variation in risk estimates. For relationships with physical activity in men within categories defined by education, roughly the same patterns emerged, but with smaller differences and without significant associations (results not shown).

**Table 4:** Risk of chronic LBP by combinations of education and physical activity in women^a^

Physical activity in leisure time (hours per week)			Duration of education (years)		
			≤9	10–12	≥13
		Total	2817	4043	2339
Light	Hard	Score	RR (95 % CI)	RR (95 % CI)	RR (95 % CI)
<1	0	1	1.00 (reference)	0.92 (0.74–1.13)	0.67 (0.48–0.93)
≥1	0	2	0.84 (0.70–1.01)	0.90 (0.75–1.08)	0.93 (0.74–1.15)
	<1^b^	3	0.88 (0.70–1.11)	0.93 (0.76–1.13)	0.83 (0.66–1.04)
	1–2	4	0.73 (0.55–0.97)	0.96 (0.78–1.18)	0.59 (0.45–0.76)
	≥3	5	1.18 (0.86–1.63)	1.06 (0.80–1.40)	0.51 (0.32–0.82)
*P,* categorical effect^c^			0.037	0.69	0.003
*P*, linear effect^d^			0.32	0.18	0.013
*P,* quadratic effect^e^			0.015^f^	0.53	0.006^g^

*LBP* low back pain, *RR* relative risk, *CI* confidence interval

^a^ Adjustment for age, work status, physical activity at work, BMI, smoking

^b^ Must have reported some hard activity

^c^ For categorical effect of physical activity, within interval for duration of education

^d^ For linear effect of score for physical activity, within interval for duration of education

^e^ For quadratic effect of score for physical activity, within interval for duration of education

^f^ Coefficient of quadratic term > 0, corresponding to a U-shaped relationship

^g^ Coefficient of quadratic term < 0, corresponding to an inverted U-shaped relationship

## Discussion

### Summary of findings

This study lends support to the notion that the risk of LBP will diminish with increasing amounts of physical activity in leisure time, probably until a certain threshold of rather extensive activity has been reached. This inverse relationship is not very pronounced, but seems to be present in both women and men, although the evidence is mainly restricted to those aged 50 years or more. It is difficult to state with certainty whether the risk of LBP actually increases when leisure time activities become very extensive, representing a U-shaped relationship. There is at least no general evidence in our study that increasing activities beyond a threshold representing 3 h of hard physical activities per week will lower the risk of LBP even further. At the same time, there are indications that relationships with heavy physical activities may differ between population groups, with extensive hard activities carrying greater risk among women with less education.

### Comparison with other studies

Numerous papers have dealt with potential associations between physical activity in leisure time and LBP. However, those most relevant for comparison with our results involve prospective studies of physical activity as a risk factor for subsequent LBP in individuals who did not initially suffer from the disorder. Among relatively large prospective studies of this kind, a study of the Nord-Trøndelag population in an earlier period [26] showed moderate inverse associations with risk of LBP in both males and females. A Danish study of twins aged 70 years or over [27] indicated a strong protective effect of strenuous physical activities. In a Finnish study of an industrial population [28], an inverse association with physical exercise was observed only in those aged 50 or over. A British study [29] did not reveal any association with the general level of physical activity in either sex. Moreover, a Finnish study restricted to individuals under 40 years old [30] found no association with non-specific LBP. Finally, in a nested case-control study in Sweden with data collected retrospectively [31], men but not women carried an increased risk of LBP with a high perceived physical load outside work.

Particular prospective studies including some individuals with LBP at baseline, but adjusting in the statistical analysis for LBP status at that stage, may also be informative. A Finnish study [32] showed an inverse association between exercise activity and a morbidity score for low back disorder. Another Finnish study [33] demonstrated a decreased risk of hospitalization due to back disorders for individuals engaged in strenuous physical activity.

Some prospective studies [27–29] have focused on a period not exceeding 2 years between baseline recording of physical activity and subsequent registration of LBP. Other studies [26, 30–32] dealt with longer periods, mostly exceeding 5 years, as in the present study. On the assumption that physical activity recorded provides information about a relatively stable situation, spanning several years, the results may then reflect long-term relationships. In these studies [26, 30–32], at least some associations between leisure time physical activity and risk of LBP were found, suggesting that long-term effects are actually present.

The exact definition of LBP has differed between relevant studies. Sometimes [26] the classic description of chronic pain has been used, requiring that pain has been experienced for at least 3 months continuously [23], as in this study. Definitions based on LBP being reported at least 30 days [27] or 7 days [28, 30, 31] during the last year may capture more cases, although they are less likely to be severe. One study [29] considered all participants reporting LBP during a 1 year period. If there is any systematic trend among results, studies applying stricter definitions [26, 27] seem to have found more associations.

Thus our general conclusion that the risk of LBP may diminish with increasing physical activity in leisure time is consistent with a large part of the relevant literature. The fact that we could only demonstrate an inverse relationship after complete adjustment in the age group above 50 years also seems to parallel earlier observations covering all ages [28] or particular age brackets [27, 30].

In addition to the cross-sectional studies [13, 15], a U-shaped relationship was reported from a prospective study [30], but only for radiating LBP. In the previous study from Nord-Trøndelag [26], risk estimates for women but not men suggested a relationship with shape similar to a J inverted horizontally, comparable to some of our results. The possibility of detecting a relationship with an approximate U-shape depends on the statistical handling of physical activity. It has been common in prospective studies to consider 3 categories of physical activity in leisure time [27–30]. The cross-sectional studies reporting U-shaped relationships used 3 [13] or 4 [15] categories in the main analysis. With very few categories, information about non-monotone relationships is easily lost in the analysis, so it is possible that underlying data sets could have revealed U-shaped relationships in even more previous studies.

### Strengths and weaknesses of the present study

Our analysis is based on a large population-based data set, with the overwhelming majority of the participants belonging to a homogeneous ethnic group [22]. Everybody in the target population was invited to participate, and participation was not associated with medical treatment of any kind. The disorder considered is limited to chronic pain localized to the lower back, providing a more specific classification of cases than in many other studies. None of the participants suffered from chronic LBP at baseline. The prospective design, with a relatively long period between collection of baseline information and registration of chronic LBP, makes it unlikely that initial stages of the disorder should affect activities reported. Information was available on several potential confounders, with occupational classification and work related activity being especially important.

The reliability and validity of the information on physical activity collected in the HUNT2 survey were assessed in a separate small study, using a retest and a check against other measures of fitness and energy expediture [34]. The information collected on hard activity in leisure time had acceptable repeatability and seemed to be a reasonably valid measure of vigorous activity [34].

As in many other studies [8], only self-reported information about back pain was available in our data. Pain intensity was not recorded and pain status in the period between the HUNT2 and HUNT3 surveys is unknown. Apart from the knowledge that back pain had been experienced continuously during a 3 month period, it is not known how the pain changed during the last year. Moreover, no information was available on changes in physical activity during follow-up. The classification of leisure time physical activity at baseline was relatively detailed, but each category considered may still include quite different activities. The categorization attempts to summarize the essential information available concerning each participant, but even the ordering of categories implied by our activity score may be inaccurate. For example, participants with extensive light activities may conceivably experience more physical strain overall than those who reported a small amount of hard activities.

The participation rate was unfortunately relatively low at end of follow-up. Participation in the HUNT3 survey has been checked against other data sources [35] and has been found to depend mainly on socioeconomic status. In the present study, the percentage of individuals not engaged in any hard physical activity in leisure time was somewhat higher in the entire cohort than among participants at end of follow-up (57 % vs. 54 % in women and 43 % vs. 39 % in men). Conversely, the entire cohort included a lower percentage of individuals engaged in hard activities ≤2 h per week in comparison to participants at end of follow-up (38 % vs. 41 % in females and 44 % vs. 47 % in males). Percentages engaged in hard activities ≥3 h per week were quite similar (5 % vs. 6 % in women and 13 % vs. 14 % in men). Non-participation at end of follow-up may thus have introduced some bias but probably not enough to alter substantially our estimates of relationships with physical activities.

### Interpretation

When particular studies have concluded that the risk of LBP depends on physical activity, most have found an inverse relationship through at least part of the activity range. This is consistent with results of controlled trials evaluating exercise for prevention of LBP [36]. There are still indications that intense physical activity carries a higher risk [8, 9], in particular activity associated with strenuous sports as rowing [37]. Heavy occupational loads involving materials handling, bending and twisting also impose a higher risk of LBP [10], but it is not clear whether similar exposure occurs regularly in leisure time, producing relationships approaching a U-shape.

In this study the shape of the relationship with physical activity in leisure time among women depended on educational background. Extensive physical activity is more common among groups in the Norwegian population with longer duration of education [38]. It is possible that women with ≥3 h of hard physical activity per week tend to engage in different exercises depending on educational background, some having a beneficial and others a detrimental effect. If so, the U-shape observed among women with basic education only may represent a genuine causal relationship. Another explanation of the interaction with education may be differential reporting of LBP. A third possibility may be residual confounding by occupational activities if such activities still vary considerably between educational groups within the occupational categories considered. In our data, women with a long duration of education had a decreased overall risk of LBP. It has previously been shown [39] that education in this population has a strong effect on disability from back pain which is not mediated by occupational class or working conditions.

Assessment of physical activity in epidemiologic studies should ideally take into account type, intensity, frequency and duration of each kind of activity [40]. It is difficult to obtain such data in large population-based studies, where physical activity is frequently evaluated using a simple one-dimensional scale. Studies of LBP prevalence using a more detailed classification of non-occupational activities [41, 42] have found different associations according to the type of activity considered. Although the evidence supporting a U-shaped relationship was not very strong overall in our study, more structured information about physical activity might reveal a more complex picture. A small study of incident LBP related to detailed accelerometer data [43] showed U-shaped associations for nearly all measures considered. Future population-based studies of risk of LBP should attempt to obtain more comprehensive information about physical activity, both in leisure time and at work, in order to assess the complete relationship.

## Conclusions

No strong overall evidence has been found for U-shaped relationships between physical activity in leisure time and risk of chronic LBP. However, engaging in 3 h or more of hard physical activity per week does not generally produce any further decrease in risk. Recommending several hours of hard physical activity per week is not justified to further reduce the risk of LBP, although particular extensive activities may still be beneficial, and especially men aged 50 years or more seem to benefit from harder exercises.

### Ethics

The work was approved by the Regional Committee for Medical and Health Research Ethics in Central Norway, and HUNT was also approved by the Norwegian Data Inspectorate. Each participant in the HUNT2 and HUNT3 surveys signed a written informed consent regarding the collection and use of data for research purposes.

## Availability of data and materials

The data set analysed belongs to a third party, the HUNT study (the Nord-Trøndelag Health Study). The authors of the current manuscript are not affiliated with the project as such, but have been given permission to analyse the data after obtaining the necessary Norwegian permits. Because of confidentiality requirements imposed by Norwegian law, data sets with information from a complete county at the individual level cannot be made public. Research groups wishing to analyse data from the HUNT study may apply to the HUNT organization (http://www.ntnu.edu/hunt) to get access. Procedures for obtaining the necessary permits are described on the web page http://www.ntnu.edu/hunt/data.

## Abbreviations

LBP: low back pain
RR: relative risk
CI: confidence interval
BMI: body mass index
